# Vectors, molecular epidemiology and phylogeny of TBEV in Kazakhstan and central Asia

**DOI:** 10.1186/s13071-020-04362-1

**Published:** 2020-10-06

**Authors:** Karlygash Abdiyeva, Nurkeldi Turebekov, Ravilya Yegemberdiyeva, Andrey Dmitrovskiy, Lyazzat Yeraliyeva, Zhanna Shapiyeva, Talgat Nurmakhanov, Yerlan Sansyzbayev, Guenter Froeschl, Michael Hoelscher, Josua Zinner, Sandra Essbauer, Stefan Frey

**Affiliations:** 1 Center for International Health, Ludwig-Maximilians-Universität, Munich, Germany; 2 National Scientific Center for Highly Dangerous Infections, Almaty, Kazakhstan; 3 Kazakh National Medical University, Almaty, Kazakhstan; 4 Scientific Center for Phthisiopulmonology, Almaty, Kazakhstan; 5 Scientific Practical Center of Sanitary Epidemiological Expertise and Monitoring, Almaty, Kazakhstan; 6 Division of Infectious Diseases and Tropical Medicine, University Hospital, LMU Munich, Munich, Germany; 7 Institute of Microbiology, Munich, Germany; 8 present Address: Bundeswehr Research Institute for Protective Technologies and CBRN Protection, Munster, Germany

**Keywords:** Tick-borne encephalitis, TBEV, *Ixodes persulcatus*, *Haemaphysalis punctata*, *Dermacentor marginatus*, Siberian subtype, Kazakhstan, Tick

## Abstract

**Background:**

In the South of Kazakhstan, Almaty Oblastʼ (region) is endemic for tick-borne encephalitis, with 0.16–0.32 cases/100,000 population between 2016–2018. The purpose of this study was to determine the prevalence and circulating subtypes of tick-borne encephalitis virus (TBEV) in Almaty Oblastʼ and Kyzylorda Oblastʼ.

**Methods:**

In 2015 we investigated 2341 ticks from 7 sampling sites for the presence of TBEV. Ticks were pooled in 501 pools and isolated RNA was tested for the presence of TBEV by RT-qPCR. For the positive samples, the E gene was amplified, sequenced and a phylogenetic analysis was carried out.

**Results:**

A total of 48 pools were TBEV-positive by the RT-qPCR. TBEV-positive ticks were only detected in three districts of Almaty Oblastʼ and not in Kyzylorda Oblastʼ. The positive TBEV pools were found within *Ixodes persulcatus*, *Haemaphysalis punctata* and *Dermacentor marginatus*. These tick species prevailed only in Almaty Oblastʼ whereas in Kyzylorda Oblastʼ *Hyalomma asiaticum* and *D. marginatus* are endemic. The minimum infection rates (MIR) in the sampling sites were 4.4% in Talgar, 2.8% in Tekeli and 1.1% in Yenbekshikazakh, respectively. The phylogenetic analysis of the generated sequences indicates that TBEV strains found in Almaty Oblastʼ clusters in the Siberian subtype within two different clades.

**Conclusions:**

We provided new data about the TBEV MIR in ticks in Almaty Oblastʼ and showed that TBEV clusters in the Siberian Subtype in two different clusters at the nucleotide level. These results indicate that there are different influences on the circulating TBEV strains in south-eastern Kazakhstan. These influences might be caused by different routes of the virus spread in ticks which might bring different genetic TBEV lineages to Kazakhstan. The new data about the virus distribution and vectors provided here will contribute to an improvement of monitoring of tick-borne infections and timely anti-epidemic measures in Kazakhstan.
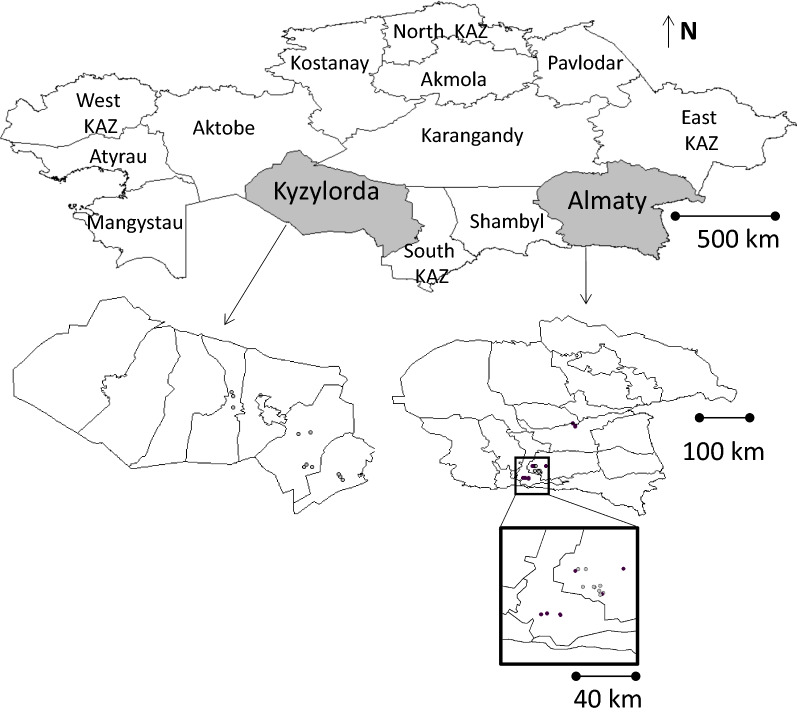

## Background

In Kazakhstan, for the first time the symptoms of tick-borne encephalitis (TBE, here Russian spring–summer encephalitis, RSSE) were described in patients from Almaty and Almaty Oblastʼ in 1935 by Steblov as described in [1]. Tick-borne encephalitis virus was isolated in Kazakhstan in 1941 by Chumakov as described in [1]. For this isolation he used the brain from a patient who had died of TBE in Almaty Oblastʼ. In 1947, Linetskaya isolated TBEV for the first time from the tick species *Ixodes persulcatus* using methods suggested by Chumakov as described in [2]. L’vov et al. [3] published in 2014 the first molecular biological data from a TBEV strain which was collected in 1977 in Almaty Oblastʼ.

TBEV is a member of the genus *Flavivirus* within the family *Flaviviridae.* Its genome consists of a single-stranded and positive-sense RNA. The genomic RNA encodes three structural proteins (C, M and E) and seven nonstructural proteins (NS1, NS2A, NS2B, NS3, NS4A, NS4B and NS5) [4]. The main subtypes of TBEV are classified according to a phylogenetic analysis of the E gene [5].

Five subtypes of TBEV are distinguished, the European (Eu-TBEV), the Far Eastern (FE-TBEV), the Siberian (Sib-TBEV), the Baikalian (Bkl-TBEV) and the Himalayan subtype (Him-TBEV) [5–8]. Each of them has its own geographical distribution. The Far Eastern subtype is mostly distributed in regions of the Far East of Russia, China and in some regions of the European part of Russia. In contrast, the European subtype occurs in countries of Europe, in the European part of Russia and occasionally in the Far East of Russia. The Siberian subtype is widespread in the territory of the European and Asian part of Russia and very rare in other European countries. The recently established Himalayan subtype was described in China in Qinghai-Tibet Plateau and the Baikalian subtype also has its own area of distribution along the Trans Baikal region, northern Mongolia, Republic of Buryatia [5, 8–12]. TBEV is transmitted by a tick bite (saliva) or rarely by consumption of infected unpasteurized or raw milk products from infected goats, sheep and cows [4, 13–15]. Different subtypes of TBEV are related to different vectors (Ixodidae). The European Subtype of TBEV is mostly transmitted by *Ixodes ricinus* and also by *Dermacentor reticulatus* while the Siberian and Far Eastern subtypes are suitable for *I. persulcatus*, although the question of vectors of the Himalayan subtype is still under evaluation [5, 16–18].

TBE is one of the most serious arboviral infections widespread on the Eurasian continent [19, 20]. It has a significant impact on public health [21]. Annually, up to 12,000 cases are registered in Europe and in Asia [22]. Kazakhstan is in close proximity to countries which are endemic for TBEV, e.g. Russia [10], China [23, 24], Kyrgyz Republic [25] and Mongolia [16, 26]. In Kazakhstan, the TBE incidence is reported to be 0.16/100,000 up to 0.32/100,000 inhabitants. About 50 cases of TBE cases are registered annually. For the last three years (2016–2018) 128 cases of tick-borne encephalitis were registered [27].

Kazakhstan has a wide range of climatic and vegetation zones with favorable conditions for different tick species, which in turn promote the prevalence of tick-borne zoonotic diseases such as TBEV [28, 29]. Characteristically TBEV endemic zones in Kazakhstan are the foothills zone and forest-steppe zone e.g. the landscape of Almaty, Almaty Oblastʼ and East Kazakhstan Oblastʼ [29]. Only a few sporadic TBE cases occur in the North and the center of Kazakhstan [27, 30, 31].

Though TBE cases in Kazakhstan are not uncommon there is a lack of data regarding the circulating TBEV strains and their molecular biological data, which would be needed to explain pathogen circulation pathways. Also, only a few concise data are available on the infection rate of TBEV in ticks, so far. Therefore, we carried out a field study to detect the minimum infection rate (MIR) in ticks and find out more details about the circulating TBEV strains in two oblasts in Kazakhstan by using RT-PCR methods, Sanger sequencing, and phylogenetic analysis.

## Methods

### Study design

Tick sampling sites were chosen according to the routes of tick collection as established by entomologists of the Scientific Practical Center for Sanitary and Epidemiological Expertise and Monitoring (SPCEEM). These routes are sites that have been observed by entomologists of SPCEEM for 10 or more years. This approach of observation is crucial for controlling the seasonal activity and abundance of tick species on the same territory.

### Tick collection

**Fig. 1:**
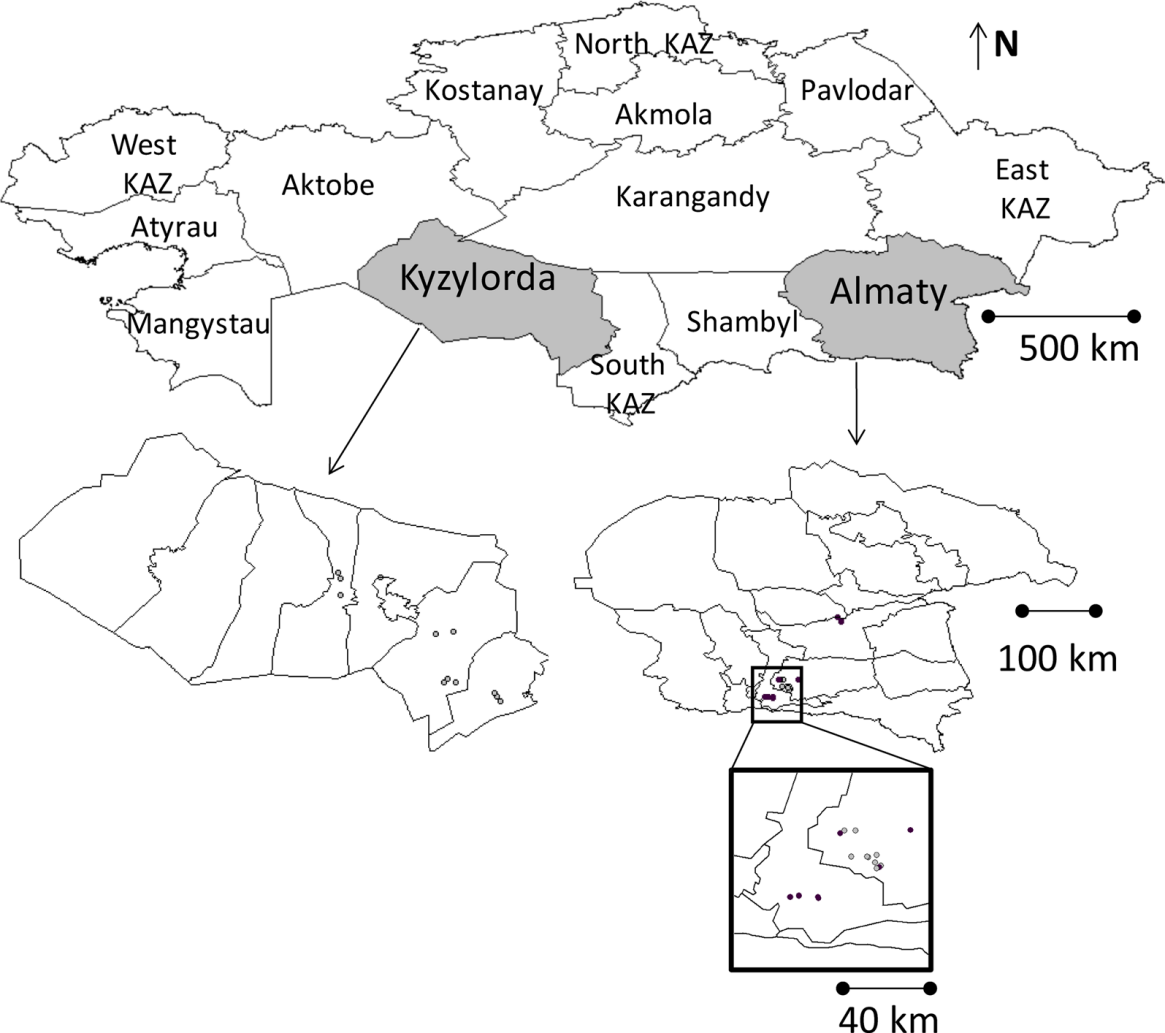
In May and June 2015 ticks were collected by flagging at 32 locations in Almaty Oblastʼ and Kyzylorda Oblastʼ. *Key*: Grey circle, TBEV-negative; Black circle, TBEV-positive

Ticks were collected by flagging at 32 locations of two oblasts (Almaty and Kyzylorda oblasts) in May and June in 2015 (Fig. 1). The collected ticks were placed into tubes, labeled according to the sampling area and frozen at −20 °C.

### Tick identification

Before testing, the ticks were thawed, morphologically identified and sorted by life stages and tick species following the official guidelines for tick identification in Kazakhstan [32–35]. The ticks (*n* = 2341) were pooled in pools of 5 adults or 10 nymphs (*n* = 501) (Table 1).

**Table 1:** The results of tick sorting (morphological identification, sorting by sex and development stage) and real-time RT-PCR examination of ticks from Almaty Oblastʼ

Region	No. of ticks/No. of pools	Tick species (No. of ticks/No. of pools)	No. of positive pools in real time PCR by sex/No. of ticks)	MIR% by tick species	MIR% by region
Talgar	505/104	*I. persulcatus* (504/103)	♂ 7/49	4.4	4.4
			♀ 15/53		
			Nymphs 0/1		
		*H. punctata* (1/1)	♂ 0/0	0	
			♀ 0/1		
Tekeli	709/145	*D. marginatus* (50/12)	♂ 0/3	2	2.8
			♀ 1/9		
		*D. reticulatus* (14/3)	♂ 0/1	0	
			♀ 0/2		
		*I. persulcatus* (610/123)	♂ 2/54	3.1	
			♀ 17/69		
		*H. punctata* (25/7)	♂ 0/4	0	
			♀ 0/3		
Yenbekshikazakh	523/113	*I. persulcatus* (79/17)	♂ 1/7	6.3	1.1
			♀ 4/9		
			Nymphs 0/1		
		*H. punctata* (444/96)	♂ 1/43	0.2	
			♀ 0/53		

*Abbreviation*: MIR, minimum infection rate

### Tick homogenization and RNA extraction

Each tick pool was homogenized with the TissueLyzer (Qiagen, Hilden, Germany) with 2 ceramic beads and 1 ml of cell culture medium. The homogenized ticks were centrifuged for 5 min at 2348 × *g*. The supernatant was used for RNA extraction according to the manufacturer instructions (QIAamp RNA viral mini kit; Qiagen). Isolated RNA was aliquoted and stored at -80 °C.

### RT-qPCRs and envelope E gene sequencing

All samples were screened for the detection of TBEV RNA. TBEV RNA was detected by reverse transcription (RT)-qPCR [36]. Five μl of TBEV RNA was amplified in a 25 μl RT-PCR mixture using the QuantiTect Virus Kit (Qiagen) with 0.2 μM of each primer (forward, 5′-GGG CGG TTC TTG TTC TCC-3′); reverse (5′-ACA CAT CAC CTC CTT GTC AGA CT-3′)) and 0.16 μM of hybridization probe (5′-6FAM-TGA GCC ACC ATC ACC CAG ACA CA-TMR-3′) according to the instructions and protocol as described before [36]. Extracted RNA of Langat virus was used as a positive control and distilled water as a negative control.

To acquire the E gene, the positive samples of the RT-qPCR were used to carry out a conventional RT-PCR with primers targeting the E gene region (1632 nt). Briefly, 5 μl of RNA was amplified with 0.2 μM of a forward primer (5′-TCT TgT gCC Tgg CTC CggT TTA Tg-3′), a mixture of two reverse primers (5′-TCC TCT gCC Tgg CTC Cgg TTT ATg-3′ + 5′-TCT TgT gCC Tgg CTC Cgg TTT ATg-3′). Into the mixture of primers and RNA, the Super Script ΙΙΙ high fidelity RT-PCR System with Platinum *Taq* DNA Polymerase (Thermo Fisher Scientific, Waltam, USA) was added. Hence the mixture was processed further in a final volume of 50 μl [37].

The initial amplification to generate cDNA was performed at 50 °C for 45 min, then a step of denaturation was carried out at 94 °C for 5 min. The amplification was conducted for 40 cycles at 94 °C for 30 s, 60 °C for 30 s and 2 min at 68 °C, followed by a final extension step at 68 °C for 10 min. The amplified products were visualized on a 1.5% agarose gel using Gel Red® (Sigma-Aldrich, Taufkirchen, Germany) under the illumination of ultraviolet light.

Products of the conventional RT-PCR were purified with the QIAquick PCR Purification Kit (Qiagen) and sequenced according to the manufacturer’s instructions with the ABI Prism Big Dye Terminator V3.1 Cycle Sequencing Kit (Thermofisher) and a 3500xl Genetic Analyzer (Thermofisher) using the initial primers used for RT-PCR amplification. Retrieved sequences were aligned using BioEdit 7.2.5 [38, 39]. Phylogenetic trees were constructed in MEGA 7 with the Maximum Likelihood method based on the Tamura 3-parameter model [38–41].

### Statistical analysis

The prevalence of TBEV in ticks was calculated using the calculation of the minimum infection rate (MIR) in the assumption that one tick in each pool was positive. MIR was analyzed according to the sampling site and tick species [37].

## Results

Overall, 2341 ticks or 501 pools of ticks from 7 sampling sites of Almaty Oblastʼ and Kyzylorda Oblastʼ were investigated (Fig. 1). Collected ticks were identified as *I. persulcatus* (*n* = 1191 adults and 2 nymphs), *D. marginatus* (*n* = 578 adults), *H. punctata* (*n* = 470 adults), *D. reticulatus* (*n* = 14 adults), *Ripicephalus turanicus* (*n* = 9 adults) and *Hyalomma asiaticum* (*n* = 77 adults).

Tick pools were screened for the presence of TBEV RNA by RT-qPCR [35]. TBEV RNA was only detected in ticks collected in Almaty Oblastʼ while the tick pools from Kyzylorda Oblastʼ were negative for TBEV. TBEV RNA from Almaty Oblastʼ was found in 48 pools with an overall MIR of 2.8%. The MIR of the different tick species was 3.9% in *I. persulcatus*, 1.6% in *D. marginatus* and 0.2% in *H. punctata* (Table 1). The MIR displayed in the geographical appearance of TBEV was 4.4% in Talgar, 2.8% in Tekeli and 1.1% in Yenbekshikazakh, respectively.

A TBEV E gene specific conventional RT-PCR was carried out with the TBEV- positive RT-qPCR nucleic acid samples, followed by sequencing [38]. The sequencing of the 1488 nt E gene open-reading frame lead to 30 E gene sequences. Eleven sequences originated from the Talgar region, 4 sequences from the Yenbekshikazakh region and 15 sequences from the Tekeli region of Almaty Oblastʼ (Table 2).

**Table 2:** Summary of TBEV E-gene sequences obtained from ticks collected in 2015

Sample no	Host	Sex	GenBank ID	Region
17	*Ixodes persulcatus*	♀	MK284381	Talgar
21		♂	MK284382	
25		♀	MK284383	
44		♂	MK284384	
45		♀	MK284385	
49		♀	MK284386	
50		♀	MK284387	
54		♂	MK284388	
64		♀	MK284389	
66		♂	MK284390	
68		♂	MK284391	
5		♀	MK284392	Tekeli
28		♀	MK284393	
33		♀	MK284394	
53		♀	MK284395	
58		♀	MK284396	
61		♀	MK284397	
99		♀	MK284398	
101		♀	MK284399	
105		♂	MK284400	
111		♀	MK284401	
113		♀	MK284402	
118		♀	MK284403	
120		♂	MK284404	
133		♀	MK284405	
137		♀	MK284406	
75		♀	MK284407	Yenbekshikazakh
77		♂	MK284408	
109	*Hyalomma punctata*	♀	MK284409	Yenbekshikazakh
112	*Ixodes persulcatus*	♂	MK284410	Yenbekshikazakh

Comparison of the Talgar E gene sequences with GenBank entries [43] revealed highest similarities at the nucleotide level (94–95%, 81–83 nt difference) to the sequence of the strain Buzuuchuk (GenBank: KJ626343) and at the amino acid (aa) level with the strain MucAr M14/10 (GenBank: AFU65175), (99%, 6 aa difference). Comparison of the Yenbekshikazakh E gene sequences with GenBank entries revealed highest similarities at the nucleotide level (94%, 84 nt difference) to the sequence of the strain Buzuuchuk (GenBank: KJ626343) and at the amino acid (aa) level with the strain MucAr M14/10 (GenBank: AFU65175), (99%, 6 aa difference).

**Fig. 2:**
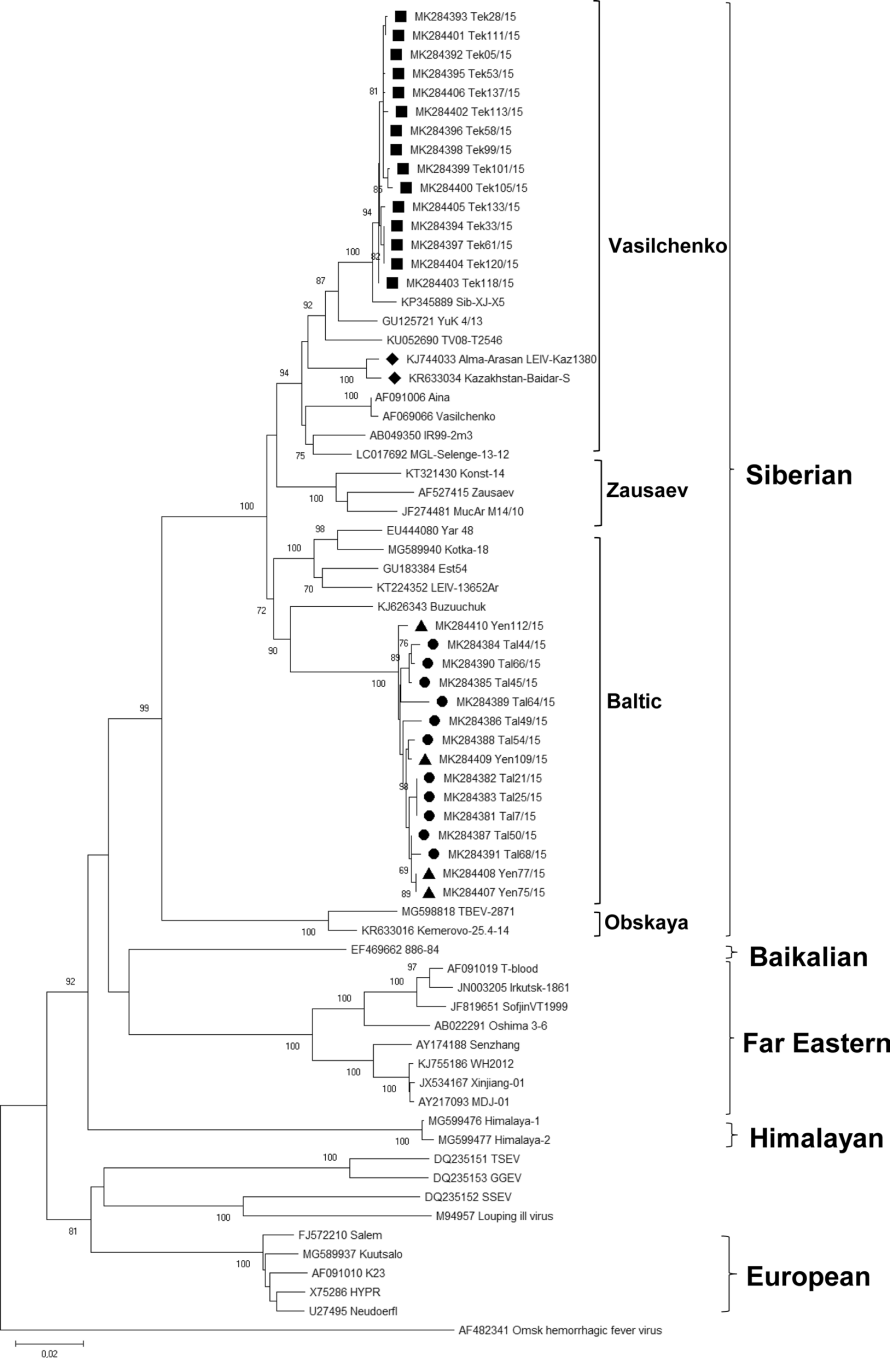
Phylogenetic tree illustrating the evolutionary relationships based on the nucleotide (nt) sequences for the envelope gene (E). The trees were constructed with the MEGA7 software package using the Neighbor-Joining method [79, 80]. The percentage of replicate trees are shown next to the branches, bootstrap test was made with 10,000 replicates [81]. Omsk hemorrhagic fever virus (GenBank: AF482341) was used as the outgroup. In the nt tree, the lineages of the Siberian subtype (Vasilchenko, Zausaev, Baltic and Obskaya) are highlighted. The newly generated sequences from Kazakhstan are marked with squares (Tekeli), circles (Talgar) and triangles (Yenbekshikazakh). The previously published TBEV sequences from Kazakhstan are marked with diamonds

**Fig. 3:**
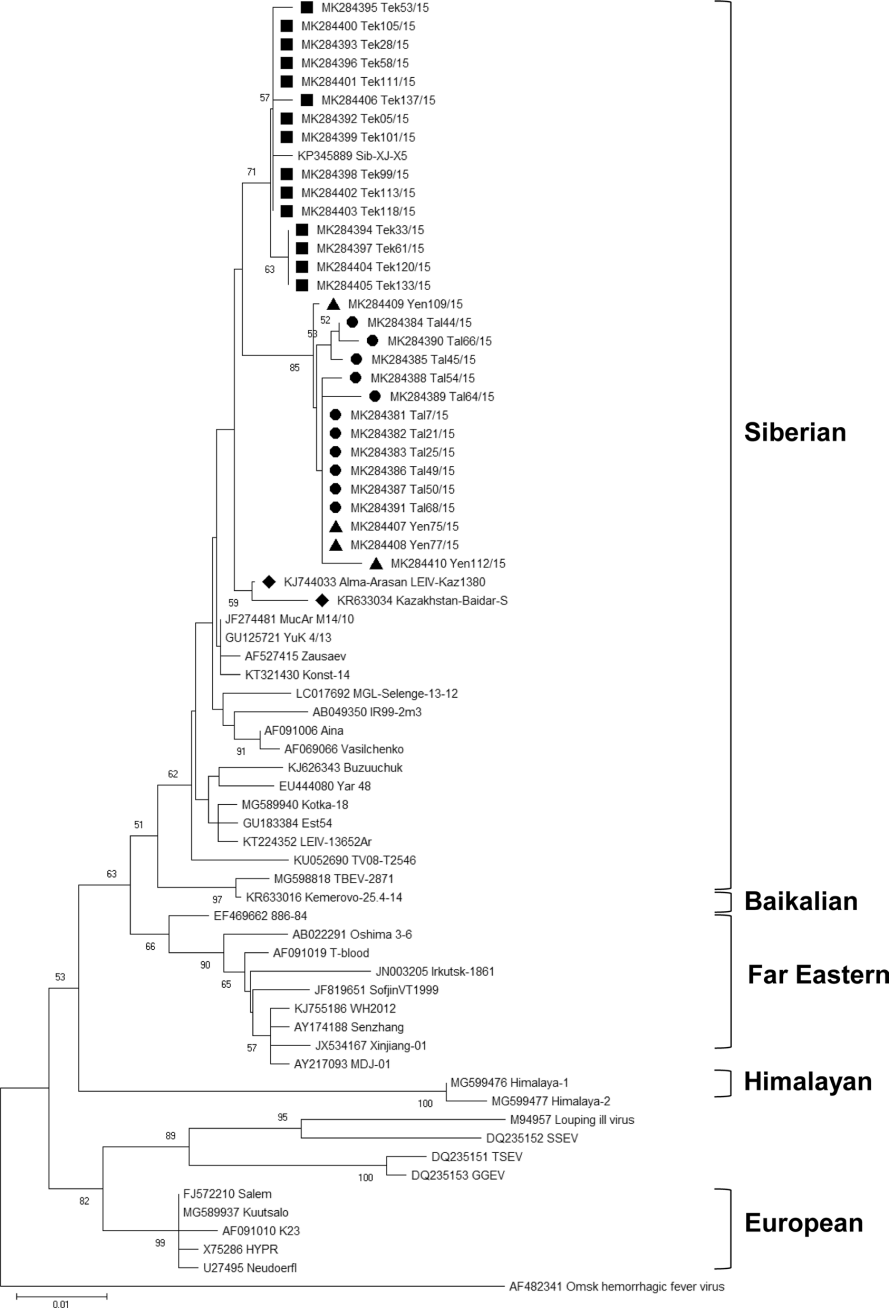
Phylogenetic tree illustrating the evolutionary relationships based on the amino acid (aa) sequences for the envelope (E) protein. The trees were constructed with the MEGA7 software package using the Neighbor-Joining method [79, 80]. The percentage of replicate trees are shown next to the branches, bootstrap test was made with 10,000 replicates [81]. Omsk hemorrhagic fever virus (GenBank: AF482341) was used as the outgroup. The newly generated sequences from Kazakhstan are marked with squares (Tekeli), circles (Talgar) and triangles (Yenbekshikazakh). The previously published TBEV sequences from Kazakhstan are marked with diamonds

The subsequent phylogenetic analyses of the E gene sequences from the 15 samples from Tekeli, the 12 samples from Talgar and the 4 samples from Yenbekshikazakh classified our strains as a Siberian subtype of TBEV on nt and aa level (Figs. 2, 3)*.* The deeper phylogenetic nt-analyses showed that our strains were clustered into two different lineages. Strains from Talgar and from Yenbekshikazakh clustered in the Baltic lineage of the Siberian Subtype. Comparison of the Tekeli E gene sequences with GenBank entries revealed highest similarities at both, the nucleotide level (99%, 15–21 nt difference) and the amino acid (aa) level (99%, 1–2 aa), to the sequence of Sib-XJ-X5, which clustered in the Siberian Subtype (Vasilchenko lineage) (Fig. 2). Phylogenetic analyses of the strains from Tekeli, Talgar and Yenbekshikazakh at the aa-level (496 aa) showed that all three strains clustered together in one clade which was divided into three sub-clusters together with other TBEV strains from Kazakhstan and the strain Sib-XJ-X5 from China (Fig. 3). In general, we observed 8 significant different aa changes within the E genes from Kazakhstan in comparison to closely related TBEV strains Zausaev (GenBank: AF527415), Konst-14 (GenBank: KT321430), MucAr M14/10 (GenBank: JF274481) and Yuk 4/13 (GenBank: GU125721) (Table 2). One position (A279) was shared by all TBEV strains from Kazakhstan including the strain from Xinjiang, Sib-XJ-X5. Four aa changes (S88, R93, I128 and I487) were only observed in the strains from Almaty city region, Talgar, and Yenbekshikazakh. One aa change in the strain Talgar and Yenbekshikazakh was detected together with the strain Sib-XJ-X5 (A313) and the strains from Tekeli revealed 2 positions (F430 and R435) which were only shared with the strain from China Sib-XJ-X5 (Table 3).

**Table 3:** Amino acid substitutions in the E-protein. Strain Zausaev was used as a reference strain

Strain/aa-pos	33	41	52	83	88	93	128	150	228	233	277	279	310	313	331	342	384	387	395	430	431	435	460	475	482	487	490	494
Zausaev	I	M	N	A	S	K	T	Y	K	Q	D	T	T	A	T	V	Y	E	K	L	A	K	L	T	L	V	M	V
Konst-14									R												T							
MucAr M14/10																					T							
YuK 4/13																					T							
Kaz-Baidar-S		I										A					H	D	R		T							
Alma-Arasan												A						D			T							
Sib-XJ-X5												A	A	T						F	T	R						
Tal17/15					G	R	I					A		T							T					I		
Tal21/15					G	R	I					A		T							T					I		
Tal25/15					G	R	I					A		T							T					I		
Tal49/15					G	R	I					A		T							T					I		
Tal50/15					G	R	I					A		T							T					I		
Tal68/15					G	R	I					A		T							T					I		
Tal44/15					G	R	I					A		T	A						T					I		
Tal45/15					G	R	I					A		T	A						T	R				I		
Tal54/15					G	R	I				E	A		T							T					I		
Tal64/15	V			V	G	R	I					A		T							T					I		
Tal66/15			T		G	R	I					A		T	A						T					I		
Yen75/15					G	R	I					A		T							T					I		
Yen77/15					G	R	I					A		T							T					I		
Yen109/15					G	R	I					A		T							T	R						
Yen112/15					G	R	I			H		A		T							T		M			I		
Tek05/15												A								F	T	R						
Tek28/15												A								F	T	R						
Tek105/15												A								F	T	R						
Tek58/15												A								F	T	R						
Tek111/15												A								F	T	R						
Tek113/15												A								F	T	R						
Tek118/15												A								F	T	R						
Tek61/15												A							R	F	T	R						
Tek33/15												A							R	F	T	R						
Tek53/15								H				A								F	T	R						
Tek99/15												A								F	T	R		S	R		T	A
Tek101/15												A								F	T	R		S	R		T	
Tek120/15												A							R	F	T	R			R		T	
Tek133/15												A							R	F	T	R					T	
Tek137/15												A				A				F	T	R						

## Discussion

During 2015 we investigated 2341 ticks/501 pools for the presence of TBEV at seven sites of two oblasts of Kazakhstan (Kyzylorda and Almaty). In Kyzylorda Oblastʼ, we investigated 136 pools and detected three species, *D. marginatus*, *R. turanicus* and *H. asiaticum*. In a previous study, six tick species were recorded in this region, i.e. *D. marginatus*, *R. turanicus*, *H. asiaticum*, *H. punctata*, *Hyalomma suspense* and *Rhipicephalus pumilio* [43]. In our study, we found that *D. marginatus* (114 pools) was dominant in comparison to the other species (*H. asiaticum* (17 pools) and *R. turanicus* (5 pools)). Following our data, we can confirm the former results that *D. marginatus* is the dominant tick species in this region [43]. This can partly be explained by the fact that the prevalence of tick species collected by flagging is affected by the peak of seasonal activity and the time of tick sampling. The peak of activity for *D. marginatus* is from April to May and *R. turanicus* usually appears later in the year [44]. However, the activity of *H. asiaticum* starts at the end of April till the end of July. Our findings reflect the time of sampling and conform to results of previous studies on the distribution of the tick species in Kyzylorda Oblastʼ [44].

In this study, the taxonomic classification of ticks was performed according morphological markers [32–35]. However, *D. marginatus* and *D. niveus* are conspecific [45–47]. In this study, both were summed up and presented as data for *D. marginatus. Rhipicephalus turanicus* (*s.s*.) has its type locality in Tashkent, Uzbekistan, and seems to be restricted to sampling sites in Central Asia, such as Kazakhstan, and southeastern Europe. For *R. turanicus* a morphological and genetical differentiation from *R. sanguineus* (*s.s*.) was provided in detail by Estrada-Peña et al. [48, 49]. Genetic markers were used to differentiate *R. turanicus* from different geographical origins and *R. turanicus* seems to be the correct species name for *R. turanicus* originating from the Palaearctic region including Kazakhstan [49–52].

All investigated ticks from Kyzylorda Oblastʼ tested negative for TBEV in the RT-qPCR. This could be explained by the absence of the main vector of TBEV, *I. persulcatus* [25, 28, 52]. The dearth of *I. persulcatus* in Kyzylorda Oblastʼ is influenced by features of the natural landscape, such as deserts and steppes with a lack of humidity [28]. In our study, we could not confirm the presence of TBEV in *D. niveus* and *H. asiaticum* or *D. marginatus* collected in Kyzylorda Oblastʼ. Regardless of the tick activity, as TBEV in Kyzylorda Oblastʼ does not appear to be endemic, it has no influence on human activities. However, the presence of TBEV was confirmed in these tick species in other regions in Kazakhstan [54–57].

From Almaty Oblastʼ (Tekeli, Talgar and Yenbekshikazakh) 365 pools were investigated and five tick species, *I. persulcatus* (243 pools), *D. marginatus* (15 pools), *D. reticulatus* (3 pools) and *H. punctata* (104 pools), were found. Our data confirm previous results on the distribution of tick species in Almaty Oblastʼ. In general, our observation shows that *I. persulcatus* and *H. punctata* prevail. In previous studies, *I. persulcatus*, *H. punctata*, *D. marginatus* and *D. reticulatus* were described for Almaty Oblastʼ [28, 44]. We can also confirm previous data showing that the tick species *I. persulcatus* and *H. punctata* are dominant in this oblastʼ [28].

TBEV RNA was revealed in the three species, *I. persulcatus*, *D. marginatus* and *H. punctata.* The study findings confirmed the results of previous studies showing that *I. persulcatus* is the main vector of TBEV [25, 28]. Our results also show that TBEV RNA can be present in *D. marginatus* and *H. punctata*. This is consistent with previously published data indicating that *D. marginatus* and *Haemaphysalis* spp. ticks could in rare cases carry TBEV [41–43]. Our data confirm that the endemicity of TBEV depends on the prevailed tick species, so that the districts of Almaty Oblastʼ where *I. persulcatus* is prevailing have the higher MIR. Only limited data on the TBEV prevalence in ticks including epidemiological data of TBE patients of the investigated areas in Almaty Oblastʼ are available.

However, the MIRs in our study reflect the prevalence of TBEV in ticks in endemic regions in the bordering countries. In Siberia, the prevalence of TBEV in ticks ranged between 0.5% and 10.2% [58], while in Europe it could reach up to 5% [58]. In bordering Mongolia, TBEV prevalence in ticks was reported in two studies to be 1.6% [16] and 1.3% [17] which is comparable to the results from Kazakhstan. In previous investigations conducted in Kazakhstan in 1970, the prevalence of TBEV in ticks reached 30% [53]. The applied RT-qPCR according to the method of Schwaiger & Cassinotti [36] has a high specificity and sensitivity [59]. It was reported that it has also higher sensitivity than conventional RT-PCR which could be affected by the amplification of the shorter fragment (approximately 68 nt) in the RT-qPCR than an amplification of a longer fragment by conventional RT-PCR (approximately 1687 nt for the E gene) [60]. Finally, we could not exclude that primers did not match perfectly the amplification sites or that the RNA structures potentially interfere with proper primer binding. This could be a reason for the observation that we had 48 TBEV-positive pools in the RT-qPCR of which only 30 samples turned out to be positive in the conventional RT-PCR.

Comparisons of E genes from Tekeli revealed that the virus strains are clustering within the branch of the Vasilchenko lineage of the Siberian subtype at the nt-level [8]. So far, most known TBEV strains from eastern Siberia cluster in this lineage. The closest related strain to the Tekeli samples was the TBEV strain Sib-XJ-X5 (99% identity, 15 nt exchanges) which was isolated from *I. scapularis* ticks in the Xinjiang region in China, bordering with Kazakhstan in the south-east.

The strains from Talgar and Yenbekshikazakh clustered within the Baltic lineage of the Siberian subtype at the nt-level [8]. Although the differences based on nt-level are quite high, the closest relative to all 15 virus sequences was the strain Buzuuchuk [61]. Buzuuchuk is an area at the Issyk-Kul Lake in Kyrgyzstan close to the border of Kazakhstan and approximately 75 km straight-line distance from Talgar.

The close relationship between the strains from Tekeli and Sib-XJ-X5 could be explained by the hypothesis that the strains were spread along bird migration routes from the Jungar Basin, Xinjiang region, China, in southwestern direction via Kazakhstan to the Arabian Peninsula. In this area, two bird flyways, the Central Asia/South Asia and East Asia/East Africa flyways are converging [62]. One of these migratory pathways is described for the Asian houbara bustards flying from China to the southwest to their wintering areas [63]. The strains from Talgar and Yenbekshikazakh also might have spread via bird migration from the southern part of the Tien Shan mountains in Kyrgyzstan. The birds might fly and spread the virus-infected ticks from one area to another through the valley of the Tien Shan mountains. However, this is not an explanation for the high genetic variability (81–83 nt difference) that occurs between the strains from Kazakhstan and the strain from Kyrgyzstan. There have been several publications showing that various tick species can be transported by birds; among them are for example studies on the tick species *I. ricinus*, which typically is the vector for Eu-TBEV [64–69]. However, the hypothesis of the spread of the virus by birds does not fully explain why the virus seems to migrate on the east–west axis, since bird migration routes predominantly follow the north–south axis. Only obstacles like higher mountain masses make birds traveling westwards such as the East Asia/East Africa flyway [62, 69]. An explanation for the spread especially along the east–west axis might be anthropogenic factors (such as human mobility or animal transportation) which were hypothesized for the strain movements of the Eu-TBEV subtype [70]. Considering the present data, the hypothesis that strains may be distributed by birds, animals or along man-made routes such as motorways or railways from the North to the South, such as for example through the route from Siberia to China or Pakistan through Kazakhstan or vice versa can be supported [66, 71, 72]. Following this, in Kazakhstan during the last years the geographical borders of the bird fauna were changed due to changes in migration of birds. Some birds in the 50 s of the last century were only seen at the borders of the southern Part of Kazakhstan, but now these have become the main bird species over the whole territory. Also, more than 20 species of trans-border birds exist within Kyrgyzstan and China. The birds nest in Kyrgyz Republic or China but fly to Kazakhstan and *vice-versa* [73].

The role of livestock is also a key factor in TBEV distribution. Ticks parasitize on mammals and could be carried by them over long distances, which would also transport TBEV-infected ticks into non-endemic areas [74]. This could be an explanation for the relationship between strains from Tekeli and China (Sib-XJ-X5). It is possible that the virus was spread by humans or animals via overland pathways from China to Kazakhstan or vice versa. The geographical region between the Kazakh and Chinese border might come into consideration because of the cultural heritage or trade routes like the movement of nomads, Mongol conquests or travelers along the ancient Silk Road [67, 75]. The same might be true for the relationship between the TBEV strains located in the North (Talgar and Yenbekshikazakh) and in the South of the Tien Shan Mountains (TBEV strain Buzuuchuk). The 15 TBEV strains from Talgar and Yenbekshikazakh are closely related to the strain Buzuuchuk. Here again, the spread could also be explained by anthropogenic factors. In particular, the genetically close relation to strains from the Baltic area could indicate the spread of this virus along trading routes along the east–west-axis such as the Silk Road or the Trans-Siberian route. Another explanation is the westward migration of the Huns or spread of the virus during the period of the Mongol Empire.

The phylogenetic tree of TBEV strains from Tekeli, Talgar and Yenbekshikazakh at the aa level showed a completely different picture. The first sub-cluster comprised the former published TBEV strains from Kazakhstan, the strain KR633034 Kazakhstan-Baidar-S, which was isolated from a blood sample of a 16-year-old patient, and the strain KJ744033 Alma-Arasan LEIV-Kaz1380 isolated from an *I. persulcatus* in the Alma-Arasan valley in the mountains in the south of Almaty city [3, 76].

The second sub-cluster comprised the strains from Talgar and Yenbekshikazakh. Interestingly the comparison of the aa sequences identified the strain MucAr M14/10 as the strain with the highest identities, with a 6 aa difference and 99% identity to the strain MK284381 from Talgar and MK284407 from Yenbekshikazakh.

The third sub-cluster comprised the strains from Tekeli. The strain from Tekeli MK284392, showed the highest identity with the strain Sib-XJ-X5, with one aa difference resulting in 99% aa identity.

At the nt-level, we can address several clusters within the Siberian subtype lineages. Comparing the strains at the aa-level, the picture is slightly different. Within the Siberian subtype, two lineages can be distinguished based on aa changes at positions 234 and 431. The Zausaev lineage is characterized by H234/A431, whereas strains of the second lineage, the Vasilchenko lineage, are characterized by Q234/T431 [20, 77]. Interestingly, all strains in this cluster (all strains from Kazakhstan, JF274481 MucAr M14/10, GU125721YuK 4/13 and KT321430 Konst-14) share the characteristics of both lineages (H234/T431) but not TBEV strain Zausaev. Our results indicate that classification of different lineages used at the nt-level cannot be used at the aa-level. It is also questionable whether the naming after Vasilchenko and Zausaev of the clusters at the aa-level still makes sense, as leads to completely different results.

In general, we observed eight significantly different aa changes within the E gene sequences from Kazakhstan in comparison to closely related TBEV strains. As a result, we conclude that the strains which are geographically originating from one region show the same characteristics at the aa-level (four unique aa changes for the strains from Talgar and Yenbekshikazakh, and two unique aa changes for the strains from Yenbekshikazakh) (see Table 3).

In previous studies, it was concluded that the presence of L and H at aa positions 206 and 234, generally characterizes the Siberian subtype [19, 78]. We confirm this for all revealed E gene aa sequences. It has been discussed that strains of the Siberian subtype with threonine at aa position 426 are typically transmitted by *I. persulcatus* [19, 79]. We also confirm this for the TBEV strains from Kazakhstan studied here.

## Conclusions

In summary, we herein describe in detail data regarding the TBEV distribution in Almaty Oblastʼ in Kazakhstan. Also, to the best of our knowledge, for the first time, we give insight about the MIR of TBEV in ticks in this endemic oblastʼ. However, to generate a complete picture about TBEV distribution, we need more genetic data in endemic regions in Kazakhstan as well as in adjacent territories. However, our data on MIR in ticks and vectors of TBEV represent a further cornerstone in improving the surveillance system in these territories. Further laboratory work with the isolation of TBEV strains from ticks collected in Almaty and other oblasts in Kazakhstan and full genome sequencing will be performed by our group.

## Data Availability

All data generated or analyzed during this study are included in this published article. The newly generated sequences were submitted to the NCBI GenBank database under the accession numbers MK284381-MK284410.

## Abbreviations

E gene: Envelope gene
MIR: Minimum infection rate
TBEV: Tick-borne encephalitis virus
PCR: Polymerase chain reaction
RT-qPCR: Reverse-transcription real time polymerase chain reaction
RNA: Ribonucleic acid
DNA: Deoxyribonucleic acid
RSSE: Russian Spring–Summer encephalitis

## References

[1] Jumatov HJ, Dmitrienko NK (1961). The peculiarities of tick-borne encephalitis natural foci in Kazakhstan.

[2] Linetskaya Y. The strains of spring summer encephalitis in Almaty oblast. Almaty: Kazakh State Medical University named V. M. Molotov (currently Kazakh National Medical University named S. D. Asfendiarov); 1949.

[3] Lvov DK, Alkhovskiĭ SV, Shchelkanov MI, Deriabin PG, Gitelman AK, Botikov AG (2014). Genetic characterisation of Powassan virus (POWV) isolated from *Haemaphysalis longicornis* ticks in Primorye and two strains of tick-borne encephalitis virus (TBEV) (*Flaviviridae*, Flavivirus): Alma-Arasan virus (AAV) isolated from *Ixodes persulcatus* ticks. Probl Virol..

[4] Kaiser R (2008). Tick-borne encephalitis. Infect Dis Clin North Am.

[5] Dai X, Shang G, Lu S, Yang J, Xu J (2018). A new subtype of eastern tick-borne encephalitis virus discovered in Qinghai-Tibet Plateau. China Emerg Microbes Infect.

[6] Demina TV, Dzhioev YP, Verkhozina MM, Kozlova IV, Tkachev SE, Plyusnin A (2010). Genotyping and characterization of the geographical distribution of tick-borne encephalitis virus variants with a set of molecular probes. J Med Virol.

[7] Demina TV, Dzhioev IP, Kozlova IV, Verkhozina MM, Tkachev SE, Doroshchenko EK (2012). Genotypes 4 and 5 of TBEV: features of the genome structure and possible scenario for its formation. Probl Virol..

[8] Tkachev SE, Chicherina GS, Golovljova I, Belokopytova PS, Tikunov AY, Zadora OV (2017). New genetic lineage within the Siberian subtype of tick-borne encephalitis virus found in western Siberia. Russia Infect Genet Evol.

[9] Zlobin V, Rudakov N, Malov I (2015). Tick-borne infections.

[10] Zlobin VI (2005). Tick-borne encephalitis in the Russian Federation: state-of-the-art and prevention policy. Probl Virol.

[11] Zlobin VI, Verkhozina MM, Demina TV, Dzhioev IP, Adelshin RV, Kozlova IV (2007). Molecular epidemiology of tick-borne encephalitis. Probl Virol..

[12] Kozlova IV, Demina TV, Tkachev SE, Doroshchenko EK, Lisak OV, Verkhozina MM (2018). Characteristics of the Baikal type of tick-borne encephalitis virus circulating in eastern Siberia. Acta Biomedica Scientifica.

[13] Caini S, Szomor K, Ferenczi E, Szekelyne Gaspar A, Csohan A, Krisztalovics K (2012). Tick-borne encephalitis transmitted by unpasteurised cow milk in western Hungary, September to October 2011. Euro Surveill.

[14] Holzmann H, Aberle SW, Stiasny K, Werner P, Mischak A, Zainer B (2009). Tick-borne encephalitis from eating goat cheese in a mountain region of Austria. Emerg Infect Dis.

[15] Balogh Z, Ferenczi E, Szeles K, Stefanoff P, Gut W, Szomor KN (2010). Tick-borne encephalitis outbreak in Hungary due to consumption of raw goat milk. J Virol Methods.

[16] Frey S, Mossbrugger I, Altantuul D, Battsetseg J, Davaadorj R, Tserennorov D (2012). Isolation, preliminary characterization, and full-genome analyses of tick-borne encephalitis virus from Mongolia. Virus Genes.

[17] Muto M, Bazartseren B, Tsevel B, Dashzevge E, Yoshii K, Kariwa H (2015). Isolation and characterization of tick-borne encephalitis virus from *Ixodes persulcatus* in Mongolia in 2012. Ticks Tick Borne Dis.

[18] Kovalev SY, Mukhacheva T (2014). Tick-borne encephalitis virus subtypes emerged through rapid vector switches rather than gradual evolution. Ecol Evol.

[19] Ierusalimsky AP (2001). Tick-borne encephalitis.

[20] Gritsun TS, Frolova TV, Zhankov AI, Armesto M, Turner SL, Frolova MP (2003). Characterization of a siberian virus isolated from a patient with progressive chronic tick-borne encephalitis. J Virol.

[21] Hayasaka D, Ivanov L, Leonova GN, Goto A, Yoshii K, Mizutani T (2001). Distribution and characterization of tick-borne encephalitis viruses from Siberia and far-eastern Asia. J Gen Virol.

[22] Donoso Mantke O, Escadafal C, Niedrig M, Pfeffer M, Working Group For Tick-Borne Encephalitis Virus C. Tick-borne encephalitis in Europe (2007). to 2009. Euro Surveill.

[23] Chen Z, Liu Q, Liu JQ, Xu BL, Lv S, Xia S, Zhou XN (2014). Tick-borne pathogens and associated co-infections in ticks collected from domestic animals in central China. Parasit Vectors.

[24] Yoshii K, Song JY, Park SB, Yang J, Schmitt HJ (2017). Tick-borne encephalitis in Japan, Republic of Korea and China. Emerg Microbes Infect.

[25] Briggs BJ, Atkinson B, Czechowski DM, Larsen PA, Meeks HN, Carrera JP (2011). Tick-borne encephalitis virus. Kyrgyzstan Emerg Infect Dis.

[26] Shapoval AN, Ulickiy LA (1980). Tick-borne encephalitis. Flaviviruses.

[27] Annual reports on selected infectious and parasitic diseases (2018). 2016–2018.

[28] Atshabar B, Burdenov LAV (2012). Atlas of bacterial and virus zoonotic infections distribution in Kazakhstan.

[29] Temirbekov JT. Tick-borne encephalitis in Kazakhstan. Almaty; 1985. p. 94–130.

[30] Kirushenko KL (1988). Tick-borne encephalitis virus Zdr Kazakhstana.

[31] Groshkova IM, Flavova MS, Popov VM, Tushnyakova MK (1959). A study on epidemiology of a tick-borne encephalitis focus in Kustanai region. Vopr Virusol.

[32] Pomerantsev BI. Ixodid ticks. Ixodidae. In: Fauna of the USSR. Arachnids. Leningrad: USSR Academy of Sciences; 1950.

[33] Philippova NA. Fauna of USSR. Arachnids. 3rd edn. Moscow: Nauka; 1977.

[34] Philippova NA. Ixodine ixodod ticks. In: Fauna of the USSR. Arachnids. 3rd edn. Moscow: Nauka; 1997.

[35] Fedorova SJ (2013). Key identification of Ixodidae at Kirghizstan. Issledovaniya zhivoy prrody Khirghizstana.

[36] Schwaiger M, Cassinotti P (2003). Development of a quantitative real-time RT-PCR assay with internal control for the laboratory detection of tick borne encephalitis virus (TBEV) RNA. J Clin Virol.

[37] Kupča AM, Essbauer S, Zoeller G, de Mendonça PG, Brey R, Rinder M (2010). Isolation and molecular characterization of a tick-borne encephalitis virus strain from a new tick-borne encephalitis focus with severe cases in Bavaria. Germany Ticks Tick Borne Dis.

[38] Zhang Z, Schwartz S, Wagner L, Miller W (2000). A greedy algorithm for aligning DNA sequences. J Comput Biol.

[39] Morgulis A, Coulouris G, Raytselis Y, Madden TL, Agarwala R, Schäffer AA (2008). Database indexing for production MegaBLAST searches. Bioinformatics.

[40] Tamura K, Stecher G, Peterson D, Filipski A, Kumar S (2013). MEGA6: Molecular Evolutionary Genetics Analysis version 6.0. Mol Biol Evol..

[41] Tamura K (1992). Estimation of the number of nucleotide substitutions when there are strong transition-transversion and G+C content biases. Mol Biol Evol.

[42] Altschul SF, Madden TL, Schäffer AA, Zhang J, Zhang Z, Miller W, Lipman DJ (1997). Gapped BLAST and PSI-BLAST: a new generation of protein database search programs. Nucleic Acids Res.

[43] Umirzakova A, Oralhanova MC (2015). About fauna of ixodid ticks (Acariformes, Ixodoidea) of Shiely region, Kyzylorda oblast. Al-Farabi Kazakh Natl Univ Bull Ecol Ser.

[44] Kalmakova M, Matjanova ASZ. About fauna of ixodid ticks - vectors in natural foci of infectious diseases. In: The problems of saving biological diversity in environment and in collections in Kazakhstan; 2016 .

[45] Estrada-Peña A, Estrada-Peña R (1991). Notes on *Dermacentor* ticks: redescription of *Dermacentor marginatus* with the synonymies of *Dermacentor niveus* and *Dermacentor daghestanicus* (Acari: Ixodidae). J Med Entomology.

[46] Filippova NA, Plaksina MA (2005). Some aspects of intraspecific variability of the closely related species of the *Dermacentor marginatus* complex (Acari: Ixodidae) as demonstration of microevolutionary process. Parazitologiya.

[47] Moshaverinia A, Shayan P, Nabian S, Rahbari S (2009). Genetic evidence for conspecificity between *Dermacentor marginatus* and *Dermacentor niveus*. Parasitol Res.

[48] Estrada-Pena A, Pfäffle MP, Petney TN, Estrada-Pena A, Mihalca AD, Petney TN (2017). Genus *Ripicephalus* Koch, 1844. Ticks of Europe and North America.

[49] Nava S, Beati L, Venzal JM, Labruna MB, Szabó MPJ, Petney T (2018). *Rhipicephalus sanguineus* (Latreille, 1806): neotype designation, morphological re-description of all parasitic stages and molecular characterization. Ticks Tick Borne Dis.

[50] Dantas-Torres F, Latrofa MS, Annoscia G, Giannelli A, Parisi A, Otranto D (2013). Morphological and genetic diversity of *Rhipicephalus sanguineus sensu lato* from the New and Old Worlds. Parasit Vectors.

[51] Nava S, Estrada-Peña A, Petney T, Beati L, Labruna MB, Szabó MP (2015). The taxonomic status of *Rhipicephalus sanguineus* (Latreille, 1806). Vet Parasitol.

[52] Bakkes DK, Chitimia-Dobler L, Matloa D, Oosthuysen M, Mumcuoglu KY, Mans BJ, Matthee CA (2020). Integrative taxonomy and species delimitation of *Rhipicephalus turanicus* (Acari: Ixodida: Ixodidae). Int J Parasitol.

[53] Dobler G (2010). Zoonotic tick-borne flaviviruses. Vet Microbiol.

[54] Karimov SK, Dernovoy AG (2001). Arboviruses and arboviral infections in Kazakhstan.

[55] Kirushenko TV, Drobishenko NI, Lvov DK, Rogovaya SGKSK, Karimov S (1980). The hosts and vectors of arboviral infectious in Ili-karatal foci. The virus ecology in Kazakhstan and in Central Asia.

[56] Hay J, Yeh KB, Dasgupta D, Shapieva Z, Omasheva G, Deryabin P (2016). Biosurveillance in central Asia: successes and challenges of tick-borne disease research in Kazakhstan and Kyrgyzstan. Front Public Health.

[57] Pakhotina VA, Vaitovich MA, Basov NY (2002). Epidemiological aspects of tick-borne encephalitis in Omsk oblast. Byull VSNTs SO RAMN.

[58] Süss J, Schrader C, Abel U, Voigt WPSR (1999). Annual and seasonal variation of tick-borne encephalitis virus (TBEV) prevalence in ticks in selected hot spot areas in Germany using a nRT-PCR: results from 1997 and 1998. Zentralbl Bakteriol.

[59] Donoso Mantke O, Aberle SW, Avsic-Zupanc T, Labuda M, Niedrig M (2007). Quality control assessment for the PCR diagnosis of tick-borne encephalitis virus infections. J Clin Virol.

[60] Dobler G, Zoller G, Poponnikova T, Pfeffer M, Essbauer S (2008). Tick-borne encephalitis virus in a highly endemic area in Kemerovo (western Siberia, Russia). Int J Med Microbiol.

[61] Demina TV, Zlobin VI, Verxozina MM, Ochirova LA, Batomunkuev AS. CVA. Analysis of the tick-borne encephalitis virus coding sequences Electron Period Publ SfedU. “Zhivie i Biokosnie Sist”. 2014; 9: 3.

[62] BirdLife International. The flyways concept can help coordinate global efforts to conserve migratory birds. 2010. https://www.birdlife.org.

[63] Combreau O, Riou S, Judas J, Lawrence M (2011). Migratory pathways and connectivity in Asian houbara bustards: evidence from 15 years of satellite tracking. PLoS ONE.

[64] Hoogstraal H, Kaiser MN, Traylor MA, Guindy E, Gaber S (1963). Ticks (Ixodidae) on birds migrating from Europe and Asia to Africa 1959–61. Bull World Health Organ.

[65] Waldenström J, Lundkvist Å, Falk KI, Garpmo U, Bergström S, Lindegren G (2007). Migrating birds and tickborne encephalitis virus. Emerg Infect Dis.

[66] Geller J, Nazarova L, Katargina O, Leivits A, Järvekülg L, Golovljova I (2013). Tick-borne pathogens in ticks feeding on migratory passerines in western part of Estonia. Vector Borne Zoonotic Dis.

[67] Adambekov S, Kaiyrlykyzy A, Igissinov N, Linkov F. Health challenges in Kazakhstan and central Asia. 2015;1–5. 10.1136/jech-2015-206251 26254293

[68] Hornok S, Flaisz B, Takács N, Kontschán J, Csörgo T, Csipak A (2016). Bird ticks in Hungary reflect western, southern, eastern flyway connections and two genetic lineages of *Ixodes frontalis* and *Haemaphysalis concinna*. Parasit Vectors.

[69] Ko S, Kang JG, Kim SY, Kim HC, Klein TA, Chong ST (2010). Prevalence of tick-borne encephalitis virus in ticks from southern Korea. J Vet Sci.

[70] Weidmann M, Frey S, Freire CCM, Essbauer S, Růžek D, Klempa B (2013). Molecular phylogeography of tick-borne encephalitis virus in central Europe. J Gen Virol.

[71] Kovalev SY, Chernykh DN, Kokorev VS, Snitkovskaya TE, Romanenko VV (2009). Origin and distribution of tick-borne encephalitis virus strains of the Siberian subtype in the Middle Urals, the north-west of Russia and the Baltic countries. J Gen Virol.

[72] Hasle G (2013). Transport of ixodid ticks and tick-borne pathogens by migratory birds. Front Cell Infect Microbiol.

[73] Kovshar AF. A field guide to the birds of Kazakhstan. Almaty; 2014.

[74] Korenberg EI, Pchelkina AA, Spitsina LN (1984). Consistent patterns in the contact of domestic animals with tick-borne encephalitis virus in the eastern part of the Russian plain. J Hyg Epidemiol Microbiol Immunol.

[75] Frachetti MD, Smith CE, Traub CM, Williams T (2017). Nomadic ecology shaped the highland geography of Asia’s Silk Roads. Nature.

[76] Pogodina VVKLS. Polytipic strains in the genofund of tick borne encephalitis. Voprosy Viruisol. 2012;57:30–6. 22905425

[77] Gould EA, Gritsun TS, Desai A (2015). The degree of attenuation of tick-borne encephalitis virus depends on the cumulative effects of point mutations. J Gen Virol.

[78] Romanova LI, Gmyl AP, Dzhivanian TI, Bakhmutov DV, Lukashev AN, Gmyl LV (2007). Microevolution of tick-borne encephalitis virus in course of host alternation. Virology..

[79] Saitou N, Nei M (1987). The neighbor-joining method: a new method for reconstructing phylogenetic trees. Mol Biol Evol.

[80] Kumar S, Stecher G, Li M, Knyaz C, Tamura K (2018). MEGA X: Molecular Evolutionary Genetics Analysis across computing platforms. Mol Biol Evol.

[81] Felsenstein J (1985). Confidence limits on phylogenies: an approach using the bootstrap. Evolution.

